# Personal comfort models based on a 6‐month experiment using environmental parameters and data from wearables

**DOI:** 10.1111/ina.13160

**Published:** 2022-11-25

**Authors:** Federico Tartarini, Stefano Schiavon, Matias Quintana, Clayton Miller

**Affiliations:** ^1^ Berkeley Education Alliance for Research in Singapore Singapore Singapore; ^2^ Center for the Built Environment University of California Berkeley California USA; ^3^ Department of the Built Environment National University of Singapore Singapore Singapore

**Keywords:** ecological momentary assessment, internet of things (IoT), machine learning, personal thermal comfort model, skin temperature

## Abstract

Personal thermal comfort models are a paradigm shift in predicting how building occupants perceive their thermal environment. Previous work has critical limitations related to the length of the data collected and the diversity of spaces. This paper outlines a longitudinal field study comprising 20 participants who answered Right‐Here‐Right‐Now surveys using a smartwatch for 180 days. We collected more than 1080 field‐based surveys per participant. Surveys were matched with environmental and physiological measured variables collected indoors in their homes and offices. We then trained and tested seven machine learning models per participant to predict their thermal preferences. Participants indicated 58% of the time to want *no change* in their thermal environment despite completing 75% of these surveys at temperatures higher than 26.6°C. All but one personal comfort model had a median prediction accuracy of 0.78 (F1‐score). Skin, indoor, near body temperatures, and heart rate were the most valuable variables for accurate prediction. We found that ≈250–300 data points per participant were needed for accurate prediction. We, however, identified strategies to significantly reduce this number. Our study provides quantitative evidence on how to improve the accuracy of personal comfort models, prove the benefits of using wearable devices to predict thermal preference, and validate results from previous studies.

**Practical implications**

In addition to demonstrating the advantages of employing wearable technology to gather subjective feedback from people, our study validates the findings from earlier research and offers quantitative evidence on how to increase the precision of personal comfort models. Our methodology and results can be used in buildings to develop and implement occupants centric controls. This enables building operators to enhance thermal comfort conditions indoors while possibly reducing the overall energy consumption of the building. We made the decision to openly publish our data so that others might use it to test various assumptions or create personal comfort models utilizing various methodologies.

## 1 INTRODUCTION

Occupant thermal comfort significantly affects how people perceive their indoor environment, and thermal dissatisfaction is an ongoing challenge. Evidence shows that approximately 40% of the 90 000 surveyed occupants in North America were dissatisfied with their thermal environment.^1^ Thermal comfort models are designed to predict comfort toward addressing this challenge. All major thermal comfort standards have models that are considered aggregate in nature.^2^, ^3^ All mainstream aggregate models aim to predict how a “typical” person or a group of people would perceive their thermal environment in terms of given environmental (e.g., relative humidity, indoor air temperature [*t*_*i*_]), and personal (i.e., clothing and metabolic rate) parameters. For example, the Predicted Mean Vote (PMV) predicts the average thermal sensation of a group of people sharing the same environment, as an outcome of the heat transfer balance model between the human body and its surrounding environment. The PMV was developed through laboratory experiments by Fanger,^4^ and is now included in both the ISO 7730:2005^2^ and ASHRAE 55‐2020 Standards.^3^

### 1.1 Limitations of aggregate models

Both the PMV and the adaptive models have several limitations when used to control the temperature in buildings,^5^, ^6^, ^7^ despite their successful adoption into international standards. (1) *Required inputs*—In real buildings, it is extremely challenging to accurately measure some input variables needed to calculate PMV, such as metabolic rate, clothing, airspeed, and mean radiant temperature.^8^ (2) *Prediction accuracy*—Even when all input variables are accurately measured, these models have poor accuracy both in predicting group and individual thermal comfort.^9^ (3) *Training*—Aggregate models do not adapt or re‐learn.^6^ They were developed using fixed and limited datasets and did not benefit from new feedback provided by people. They do not learn and adapt to specific conditions.^5^ (4) *Limited inputs*—Aggregate models only use a small set of input variables. They do not use variables, such as skin temperature (*t*_*sk*_), heart rate (*HR*), age, or health status, that may affect the thermal perceptions of people.^5^

### 1.2 The emergence of personal comfort models

Personal comfort models challenge the *one‐size‐fits‐all* approach of aggregate models. Instead of an average response from a group of people, a single model is trained and tested for each participant. Personal comfort models are, however, not limited to predicting one person's thermal preference. Their aggregated outputs can be used to predict the thermal preference of a large group of people sharing the same environment.^5^ Since their introduction, personal comfort models have been expanded to leverage data collected using a wide array of sensors, including portable sensors and devices,^10^, ^11^ building management systems,^12^, ^13^ personal comfort systems,^14^ as well as onboard sensors in wearable devices and smartphones. This network of sensors can remotely and non‐intrusively measure, log and store spatiotemporal environmental and physiological data.

Wearable devices have increased the viability of personal model development due to the use of *physiological sensors* to improve model accuracy. For example, skin temperature ($t_{\mathrm{sk}}$) reflects the vasomotor tone^15^ while heart rate correlates with activity levels. This is supported by previous research that has shown that the use of $t_{\mathrm{sk}}$ as an independent variable can improve the prediction accuracy of thermal comfort models.^16^, ^17^, ^18^, ^19^, ^20^, ^21^ In certain applications, $t_{\mathrm{sk}}$ may be even determined using non‐contact sensors like infrared.^22^, ^23^, ^24^ However, it is essential to emphasize that non‐contact sensors are less accurate than those that are in direct contact with the skin; they can only monitor $t_{\mathrm{sk}}$ from body areas that are in the line of sight to the camera and are expensive to install.^6^ They, however, do not require having a sensor to be worn by people. Experimental methodologies collecting $t_{\mathrm{sk}}$ are common and iButtons sensors are often used. They can accurately measure and log $t_{\mathrm{sk}}$_._ ^25^, ^26^, ^27^ Currently, most smartwatches on the market can measure *HR* with sufficient accuracy for thermal comfort research; however, none incorporate sufficiently accurate skin temperature sensors.^18^

### 1.3 Limitations of personal comfort models

Despite the momentum of personal comfort models, there are still several unknowns and limitations as outlined in a recent review.^28^ This analysis pinpoints a lack of diversity in space types, climates, and conditions used to train personal comfort models. The review showed that only 3 out of 37 studies selected for analysis included data collection outside office spaces or lab‐based thermal chambers used to emulate an office environment.^28^ Another limitation is that there was a wide range of the amount of longitudinal data collected in the studies, with anywhere between 8 and 416 points collected per person. Researchers placed little emphasis on whether the length and data amount were exhaustive in capturing the predictability of an individual. In addition, in personal comfort model experiments, it is not common or easy to log and measure information about the participant's dynamic personal factors such as clothing or activity levels.^29^ Addressing the lack of diversity and the amount of data is not easy due to experimental constraints.

One of the biggest challenges that researchers currently face is recording how people perceive their thermal environment over a long period of time while minimizing the fatigue of completing a Right‐Here‐Right‐Now (RHRN) thermal comfort survey. To partially solve this issue, Kim et al.^30^ tried to infer occupants' thermal preferences by analyzing specific behaviors, such as turning on and off heating and cooling devices. They then coupled these data with environmental readings to infer a user's preferences without them having to complete a survey. However, thermal actions may be triggered by other reasons besides thermal discomfort; for example, Kim et al.^30^ found that users turn on the heating element in their chair to mitigate back pain.

### 1.4 Improving personal comfort models through larger and more diverse longitudinal data

To address the limitations mentioned above, an emerging methodology focuses on the use of wearable devices to collect physiological data and act as the subjective feedback collection interface. This method builds upon research in the area of Ecological Momentary Assessments (EMA), a form of collecting subjective information in diverse field‐based settings.^31^ A style of this methodology emerging as a popular way to reduce the incidence of survey fatigue is micro‐EMA, in which smartwatches are used to prompt a research participant to leave feedback in a fast and time‐efficient manner.^32^ Micro‐EMA has been shown to deliver higher response rates with a lower burden on research participants than a smartphone or computer‐based survey.^33^ To build upon this foundation and help solve the issue of collecting perception data from people, our team has contributed to the development of the micro‐EMA Cozie project that targets indoor occupant data collection.^34^, ^35^ Cozie is an open‐source application that one can install on Fitbit (Versa 2 and Ionic) or Apple smartwatches. The platform has been utilized in previous studies to test the implementation and modelling of smartwatch‐based subjective data collection,^36^, ^37^, ^38^ study thermal preference, imbalanced classes,^39^ and create personal comfort models using building information model components as inputs.^40^ One can find more information about Cozie and the official documentation at https://cozie.app and https://cozie‐apple.com. Cozie allows people to conveniently complete an RHRN survey via their smartwatches. Subjects' perceptions, preferences, and behaviors collected via Cozie can then be coupled with environmental data collected from wireless sensing devices and physiological data collected by the smartwatch.

### 1.5 Aim and objectives

Our research aims to resolve gaps in personal thermal comfort models by collecting field‐based thermal preference data. Our methodology is designed to enable us to address the following questions with resulting novel insights:

- How many data points per user must be collected to develop a reliable and robust personal comfort model? We collected data for 180 days resulting in more feedback responses per person (up to 1080) than in any previous study.^28^
- Are environmental and physiological data sufficient to train personal thermal comfort models while minimizing the impact on users? The methodology of this paper utilizes a novel framework of simple‐to‐use non‐intrusive techniques to collect physiological, environmental, and geospatial data using smartwatch‐based micro‐EMA.
- Can increasing the diversity of space types and conditions improve the accuracy of personal comfort models? How can different variables contribute to the overall model accuracy? This study is designed to collect data from diverse spaces, including the participants' homes, where there is a lack of data in previous studies. In addition, this paper is novel in accurately monitoring whether the RHRN was completed during transitory conditions.

In addition, we decided to publicly share our data so other people can use it to test different hypotheses or develop personal comfort models using a different methodology.

## 2 METHODOLOGY

**FIGURE 1:**
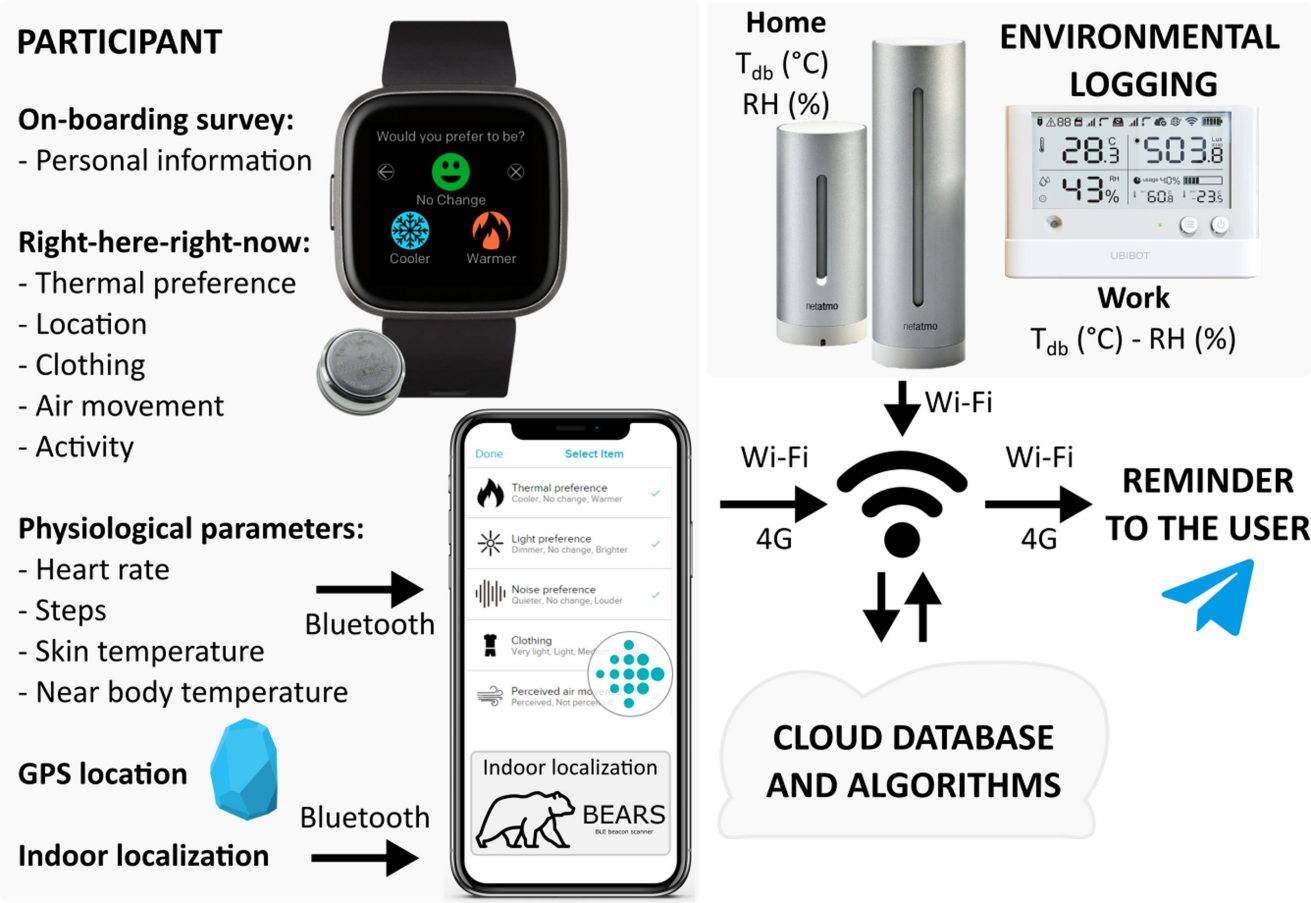
Methodology used to collect data in our study. Participants answered the RHRN surveys using the Fitbit Cozie clock face. Physiological data and RHRN responses were first sent to the Fitbit companion application and then synced with a cloud database. The *HR* data were downloaded from the Fitbit accounts. $t_{\mathrm{sk},w}$ and $t_{\mathrm{nb},w}$ were measured using two iButtons which were installed on the Fitbit wristband. Indoor location was monitored using two BLE beacons communicating with the BEARS Android application when each participant's phone was in their proximity. Environmental data were uploaded to the cloud database using Wi‐Fi. Finally, participants were reminded to complete the RHRN surveys using Telegram, a messaging application.

We collected subjective responses and physiological data from human subjects using wearable devices, personal data using surveys, and environmental data using data loggers. We then applied supervised machine learning algorithms to train personal thermal comfort models for each study participant. Thermal preference votes from the RHRN survey (i.e., Q.1 Cozie Survey—Thermal preference—please see Section 2.4) were utilized as the ground truth labels for model training and evaluation. The methodology and sensors we used to measure and log data are summarized in Figure 1, while a flowchart depicting the methodology we used to analyze the data is shown in Figure A.2. The human subject experiment for this study was approved by the University of California Berkeley IRB (Institutional Review Board: 2020‐01‐12899). We compensated participants who completed the study with gift vouchers for a total amount of SGD 400.

### 2.1 Subjects

Participants were recruited through online posting. The inclusion criteria were that the participant must: have lived for at least 3 months in Singapore, be at least 21 years old, and be fluent in English. Personal information (e.g., sex, age, and education) about participants was collected using a web‐based survey at the beginning of the study.

### 2.2 Wearable sensors

Each participant received a Fitbit Versa (v1 or v2) and was asked to wear it daily for the whole duration of the study.

To measure and log wrist skin temperature ($t_{\mathrm{sk},w}$) and wrist near body temperature ($t_{\mathrm{nb},w}$) we installed two iButtons, model DS1925, on the Fitbit wristband. One iButton was installed on the inner side of the wristband and measured $t_{\mathrm{sk},w}$ in the front part of the wrist. The other was installed above the watch display and was used to measure $t_{\mathrm{nb},w}$. Figure A.1 shows the exact location of where the iButtons were installed.

More information about the rationale on why we used Fitbit and iButton can be found in Section 1 of the Appendix.

Participants were asked to complete the RHRN no sooner than 10 min after either wearing the Fitbit or changing clothes or activities. This further limits the error in the measurement of $t_{\mathrm{sk},w}$ and ensured that they did not complete an RHRN survey during a transitory.

### 2.3 Environmental sensors

**FIGURE 2:**
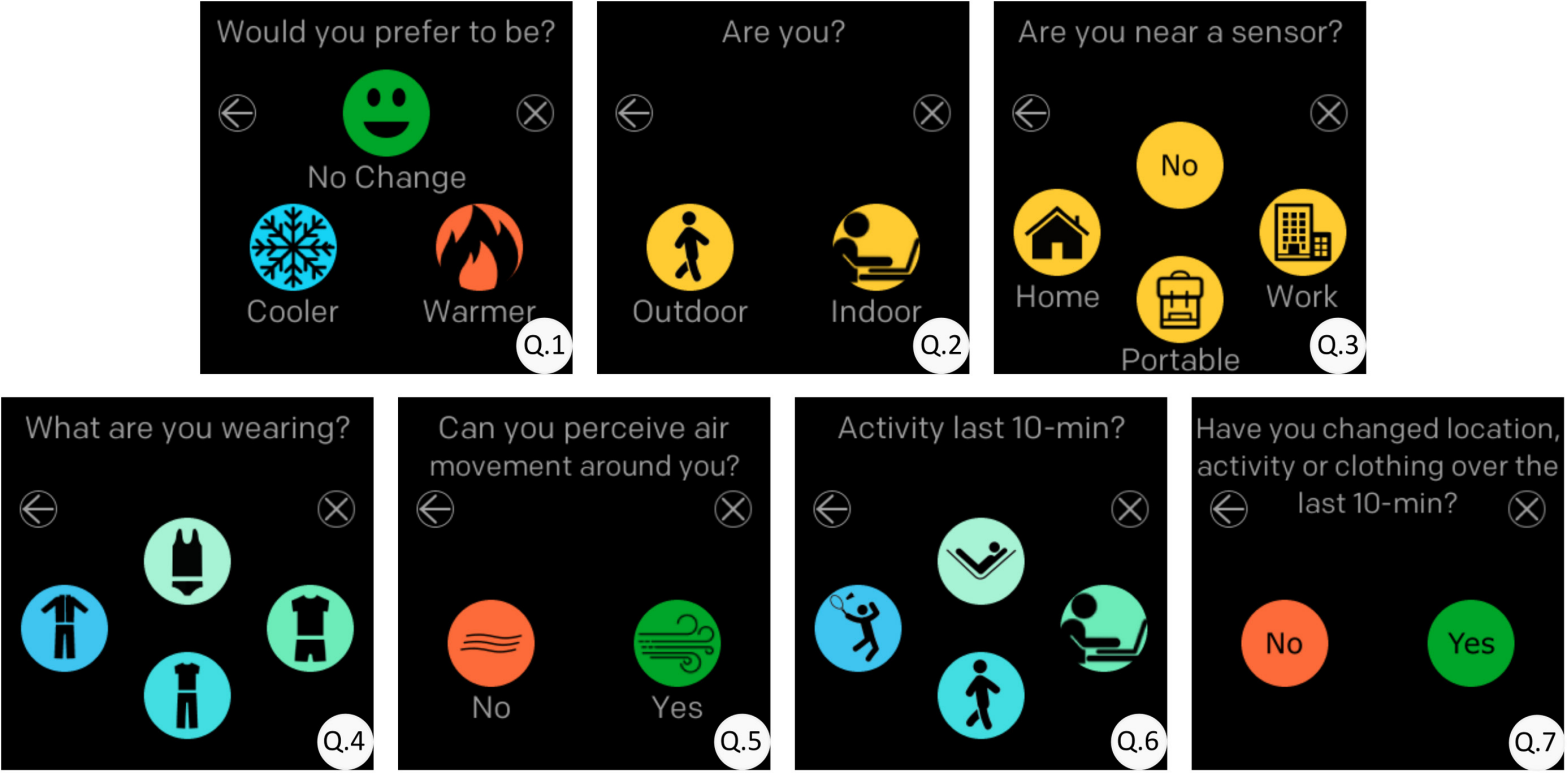
Right‐Here‐Right‐Now (RHRN) survey questions displayed using the Cozie clock face

Environmental data were monitored and logged using three sensors. One was installed in the room of their house, where they spent the majority of their time indoors. This room corresponds to the “Home” location in question three of the Cozie survey as shown in Figure 2. Another was used to measure and log $t_{i}$ and relative humidity at the participant's workplace. This room corresponds to the “Work” location in the Cozie survey. The workstation could be in their office or home if they were working from home. Finally, the third sensor on a bag/backpack of their choice. Participants were instructed to select “Portable” in question three when within a 2 m radius of this sensor. Detailed information about each sensor used is presented in Section 1 of the Appendix and Table A.1.

### 2.4 Surveys

Participants were asked to complete, on average, a total of 42 RHRN surveys per week over a period of 180 days using the Cozie clock face. Figure 2 shows the flow of questions that were included in the RHRN survey.

Q.1—“Would you prefer to be?” assesses the thermal preference using a three‐point scale. Q.2—“Are you?” logs if participants completed the survey either indoors or outdoors. Q.3—“Are you near a sensor?” determines if a participant is in proximity to one of the three environmental sensors. Q.4—“What are you wearing?” participants reported their clothing level using a 4‐point ordinal scale. Q.5—“Can you perceive air movement around you?” assesses if the air surrounding the participant was still. Q.6—“Activity last 10‐min?” participants reported their activity level over the last 10 min. Q.7—logged if the survey is answered during a transitory situation or in a near “steady‐state” environment.

The questions flow was always displayed in the same order. A custom‐made algorithm analyzed real‐time environmental data and occupants' indoor location that was logged by an application we developed. Participants received a message when in the proximity of the two environmental sensors, and they had completed less than 10% of the total RHRN surveys in those environmental conditions.

### 2.5 Weather data

Weather data were obtained from the Singapore Government website that provides 1‐min interval data.^41^ Weather data was merged with the GPS information collected by the Cozie app and answers to question two of the RHRN survey.

### 2.6 Data analysis

The source code we used to analyze the data and the full dataset are publicly available at this URL: https://github.com/FedericoTartarini/dorn‐longitudinal‐tc‐study.

#### 2.6.1 Data preparation

Participants completed surveys while performing a wide range of activities, wearing different clothing, being in multiple locations, and being exposed to a broad range of environmental conditions.

We aimed to develop a personal thermal comfort model for each participant, which could potentially be used to better control and operate buildings. Consequently, we decided to exclude the responses that participants provided: (i) while exercising, (ii) when not in the proximity of either of the environmental sensors provided (answered “No” to Q.3), (iii) during a transitory situation (answered “Yes” to Q.7), (iv) when outdoors, and (v) while not wearing the smartwatch correctly. The rationale behind our decisions was that personal comfort models could mainly be used indoors to improve thermal comfort conditions where environmental conditions can be controlled. We provide a detailed description of how we implemented the above‐mentioned selection criteria in Section 2 of the Appendix.

#### 2.6.2 Supervised machine learning algorithms

We used seven supervised machine learning classifiers to predict thermal preferences: Logistic Regression (LR), Random Forest (RDF), Extreme Gradient Boosting (XGB), Support Vector Machine (SVM), K‐Nearest Neighbors (KN), Gaussian Naive Bayes (GNB), and Multi‐Layer Perceptron (MLP). We used the Kruskal–Wallis *H*‐test to test the null hypothesis that the population median of all the groups is equal. The Kruskal–Wallis *H*‐test was used since the ANOVA assumptions were not satisfied, and it is a non‐parametric version of ANOVA. The rejections of the null hypothesis do not indicate which of the groups differs. Comparisons between groups are required to determine which groups are different.

#### 2.6.3 Training data size

One of our objectives was to determine how the number of training data points would affect the model accuracy. This has practical applications since it would inform us of the minimum required number of RHRN to be collected from each participant. The hypothesis is that a higher number of data for each participant would lead to more accurate results. To test this, we randomly selected 100 data points for testing and then trained the models using the first 42 RHRN surveys (approximately 1 week of data) each participant completed. We then iteratively trained a new model for each increment which comprised additional 84 training data points.

#### 2.6.4 Independent variable selection

The independent variables we used to train our models are shown in Table 1. Each column represents a sub‐set of variables and each row the respective model. The variables were grouped as follows: environmental—outdoor air temperature, outdoor humidity ratio, indoor air temperature ($t_{i}$), and humidity ratio indoors (*W*_*i*_); clo–met—self‐reported clothing and activity as explained in Section 2.4; wearable—location, heart rate (*HR*), wrist skin temperature ($t_{\mathrm{sk},w}$), and wrist near body temperature ($t_{\mathrm{nb},w}$); time—hour of the day, weekday or weekend, and day of the week.

**TABLE 1:** Independent variables used to train the respective model

	Variable sets					
Model	env	time	wear	clo–met	env‐hist	wear‐hist
Thermal preference PCM	x	X	x			
Thermal preference PCM clo–met	x	X	x	x		
Thermal preference PCM clo–met hist	x	X	x	x	x	x

*Note*: We used the following abbreviations in the table: self‐reported clothing and activity (clo–met), environmental (env), wearable (wear), and historical (hist).

We also computed some variables (*hist*) to take into account how thermal history may have influenced how participants perceived their environment at the time of completing the RHRN survey. For each of the time‐series data included in either the *environmental* or the *wearable* variable sets, we calculated the following additional variables: exponentially weighted moving average and gradient over a 20 and 60 min period preceding the survey. The average and gradient for the weather data were calculated using timeframes of 1 and 8 h.

We used the SHapley Additive exPlainations (SHAP) method to determine how much each variable influences the output of the model. The primary idea behind Shapley's value‐based explanations of machine learning models is to divide the credit for a model's output among its input variables using fair allocation outcomes from cooperative game theory.^42^, ^43^ The use of the SHAP approach allowed us to understand and interpret how and why our complex models made specific predictions.

We included *env*, *time*, and *wearable* in all models since previous research has demonstrated that the inclusion of these variables into personal comfort models significantly increases their prediction accuracy.^18^ We, therefore, decided only to test whether the use of historical and self‐reported clothing and activity would have improved the prediction accuracy in our case.

We have shared the data we collected publicly so other researchers may test different hypotheses or use a different approach from the one described in this paper.

Including indoor air temperature ($t_{i}$), wrist skin temperature ($t_{\mathrm{sk},w}$), and wrist near body temperature ($t_{\mathrm{nb},w}$) in all models may introduce multicollinearity. The environment to which a person is exposed, the clothing they wear, and the actions they perform, together which several other factors that affect how indoor air temperature ($t_{i}$), wrist skin temperature ($t_{\mathrm{sk},w}$), and wrist near body temperature ($t_{\mathrm{nb},w}$) are correlated. We, therefore, decided to keep them all in the models since they allowed us to potentially capture all the above‐mentioned interactions that cannot be measured but still play a significant role in how people perceive their thermal environment. For example, the near‐body temperature may approximate the air temperature when a person is exposed to elevated air speeds. On the other hand, it will be more influenced by the skin temperature when the person is resting and the air in the room is still. It is worth mentioning that Apple in their latest smartwatch, the Apple Watch 8 released in October 2022, also included two temperature sensors, one that measures the skin temperature and one below the screen to isolate the body temperature from the outside environment. Apple claims that this allows them to get a more accurate estimate of the variables that they want to predict.^44^

### 2.7 PMV estimation

We used the measured environmental variables and personal factors, qualitatively logged by the participants to calculate the PMV using the following assumptions. The activity levels reported by the participants were mapped using the following values resting = 0.8 met, sitting = 1.1 met, and standing = 1.4 met. While reported clothing values were mapped as follows very light = 0.3 clo, light = 0.5 clo, medium = 0.7 clo, and heavy = 1.0 clo. These numbers were determined by asking each participant which clothes on average they wore when selecting one of the above options. The mean radiant temperature was assumed to be equal to $t_{i}$_._ ^45^ The relative airspeed value was calculated assuming the airspeed to be equal to 0.1 m/s and using the self‐reported activity levels. We are fully aware that these assumptions have limitations and do affect PMV prediction accuracy; however, similar assumptions have been previously used in thermal comfort research.^30^ Finally, we mapped the PMV values into thermal preference votes using the following assumptions: “Warmer” for PMV <1.5, “Cooler” for PMV >1.5, and “No Change” for −1.5 ≤ PMV ≤ 1.5. This is the same assumption made by Fanger who considers dissatisfied those people who reported their absolute value of thermal sensation to be either 2 or 3.^4^ This is based on the assumption that, for example, people who have a thermal sensation of “Warm” or “Hot” is highly probable that they may want to be “Cooler.” In this paper, we did not draw conclusions on the accuracy of the PMV model, but we only used it as a benchmark value to assess the accuracy of the thermal personal comfort models.

#### 2.7.1 Evaluation criteria

The model prediction accuracy was evaluated using the following metrics: F1‐micro, F1‐macro, and Cohen's kappa. We calculated all these metrics for a more precise interpretation of the results, however, we only reported the F1‐micro scores unless there was a significant disagreement between the prediction accuracy scores of different metrics. F1‐micro ranges between 0 and 1 where 1 represents the optimal prediction value. F1‐micro measures the prediction accuracy and gives equal importance to precision (true positives divided by all positive results) and recall (true positives divided by the number of samples that should have been identified as positives). In multilabel classification, (i.e., in our case since thermal preference assumes three values) the F1‐micro is calculated globally across all classes.

#### 2.7.2 Training and testing

Hyper‐parameters optimization is done using a random search and 5‐fold cross‐validation. We tested 10 random combinations of hyper‐parameters in each of the 5‐fold, and the best performing model, in terms of objective metric as specified in Section 2.7.1, is chosen. Table A.2 shows the parameters chosen for training the models and performing the random search. We repeated this entire process 100 times for each model.

## 3 RESULTS

The longitudinal study commenced in April 2020 and ended in December 2020 in Singapore. A total of 20 participants (10 males and 10 females) took part in our study. Key information about each participant is presented in Table 2.

**TABLE 2:** Information about the subjects

ID	Sex	Age	Education	BMI (kg/$m^{2}$)
1	M	38	Doctoral degree	23.51
2	M	36	Doctoral degree	29.40
3	M	30	Doctoral degree	25.54
4	F	40	Master's degree	18.29
5	M	31	Doctoral degree	25.39
6	M	44	Doctoral degree	21.22
7	F	30	Bachelor's degree	25.93
8	M	35	Doctoral degree	25.10
9	F	24	Master's degree	23.24
10	M	24	High school graduate	23.05
11	F	29	Master's degree	20.20
12	M	34	Doctoral degree	28.20
13	M	31	Bachelor's degree	25.34
14	M	35	Bachelor's degree	23.03
15	F	33	Doctoral degree	18.34
16	F	26	Bachelor's degree	20.45
17	F	36	Doctoral degree	18.37
18	F	26	Bachelor's degree	22.04
19	F	24	Bachelor's degree	16.44
20	F	32	Doctoral degree	20.96

### 3.1 Dataset preparation and cleaning

Participants completed a total of 22212 RHRN. Of the total surveys collected, participants completed 2% of them while exercising, 6% while outdoors, and 12% while in transitory conditions. These surveys were not included in the data analysis as previously explained in Section 2.6.1.

**FIGURE 3:**
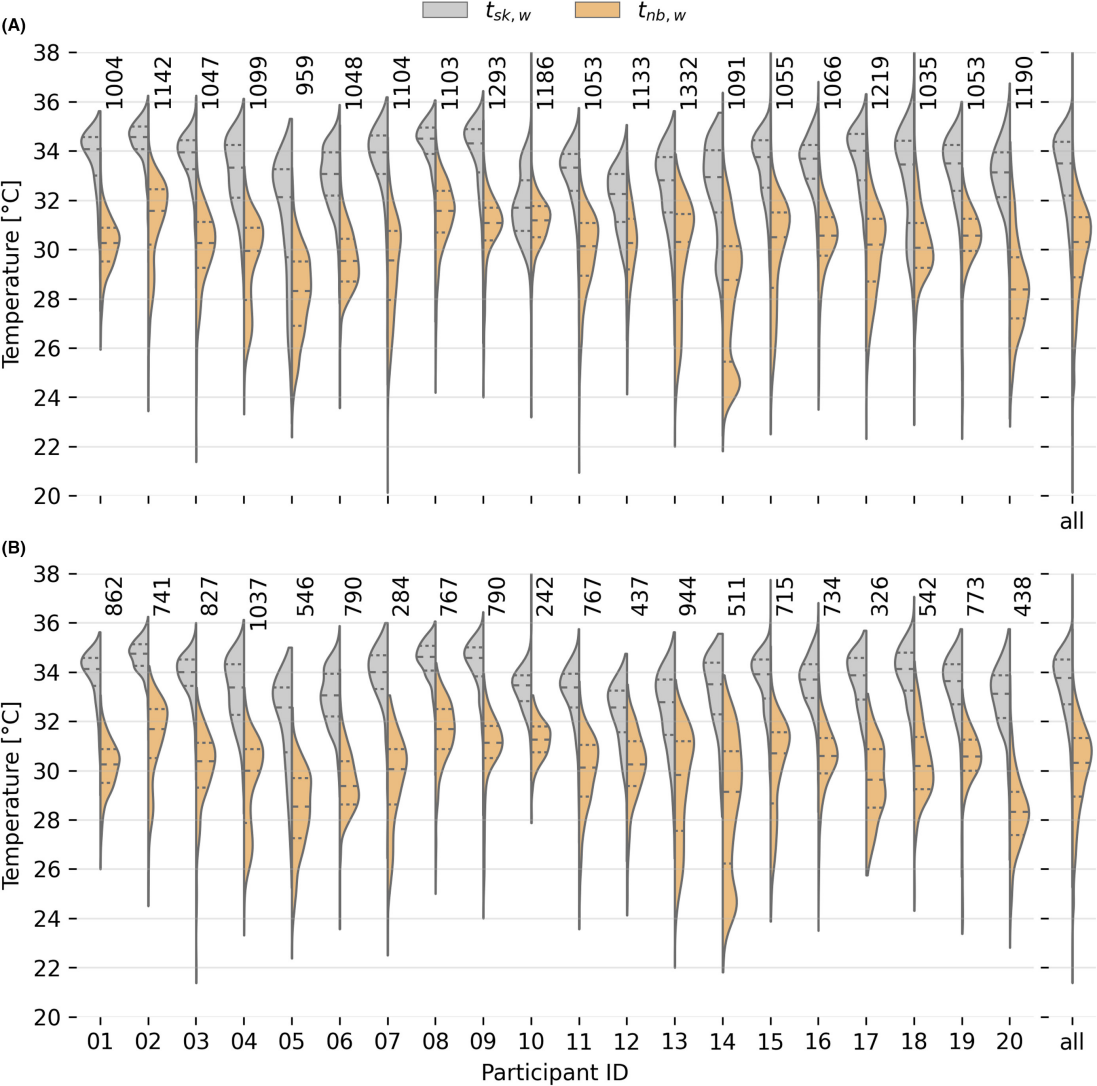
Wrist skin temperature ($t_{\mathrm{sk},w}$) and wrist near body temperature ($t_{\mathrm{nb},w}$) measured when the participants completed the RHRN survey. (A) Shows all the data collected from the participants while (B) shows the sub‐set of the original dataset that was used in the data analysis. The inclusion criteria we used to filter the original dataset are detailed in Section 3.1. The number above each violin plot is the number of RHRN surveys completed by each participant.

The $t_{\mathrm{sk},w}$ and $t_{\mathrm{nb},w}$ data we measured while the participants completed the RHRN are depicted in Figure 3A. In approximately 97% of the total completed surveys, the value of $t_{\mathrm{sk},w}$ was higher than $t_{\mathrm{nb},w}$. This result was expected since the maximum value of $t_{i}$ that participants experienced throughout the study never exceeded 34°C. For example, the delta between $t_{\mathrm{sk},w}$ and $t_{\mathrm{nb},w}$ in participant 10 was as low as −0.7°C, while the average value across all participants was −3.2°C. We consequently remove the data using the methodology detailed in Section 2 of the Appendix. This removed more than 15% of the total number of surveys collected by the following participants 05, 10 (73% excluded), 12, 14, and 18.

This sub‐set of the original dataset, which included 13 073 survey responses, was used in the data analysis. The filtered number of surveys for each participant is shown in Figure 3B.

### 3.2 Dataset overview

**FIGURE 4:**
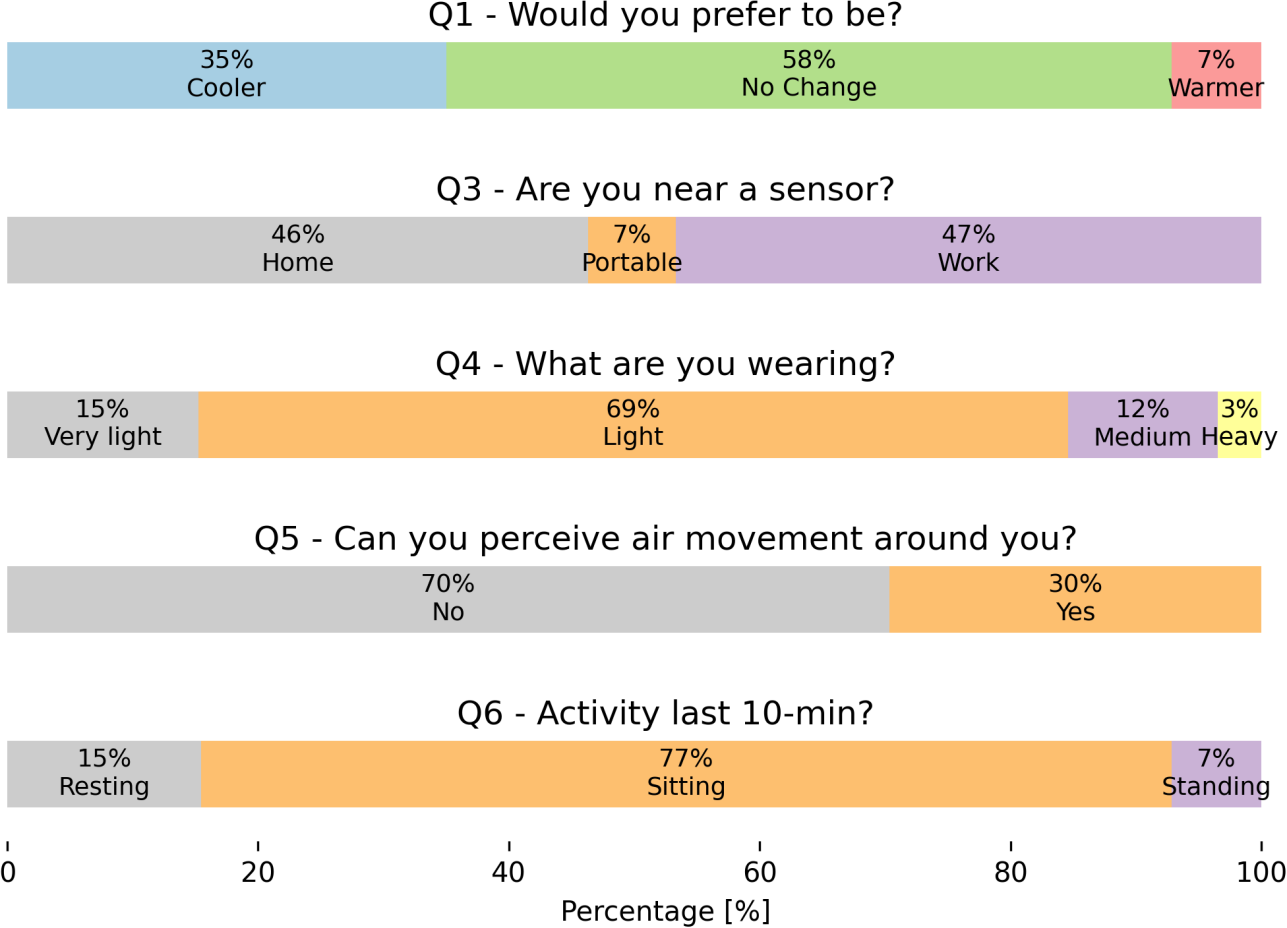
Distribution of the answers provided by all the participants.

The 13 073 survey responses are summarized in Figure 4. Votes in Q.1—“Thermal preference” were mostly “No Change” (58%) followed by “Cooler” (35%). This study took part during the COVID‐19 pandemic, and most of the participants had to work from home for the whole study duration. Participants in their homes had full control of the air‐conditioning set‐point and could use electric fans to increase airspeed in their surroundings.

Most of the participants reported being involved in sedentary activities in 77% of the cases. Participants perceived air movement only less than 30% of the time, and 69% of them wore “Light” clothes.

**FIGURE 5:**
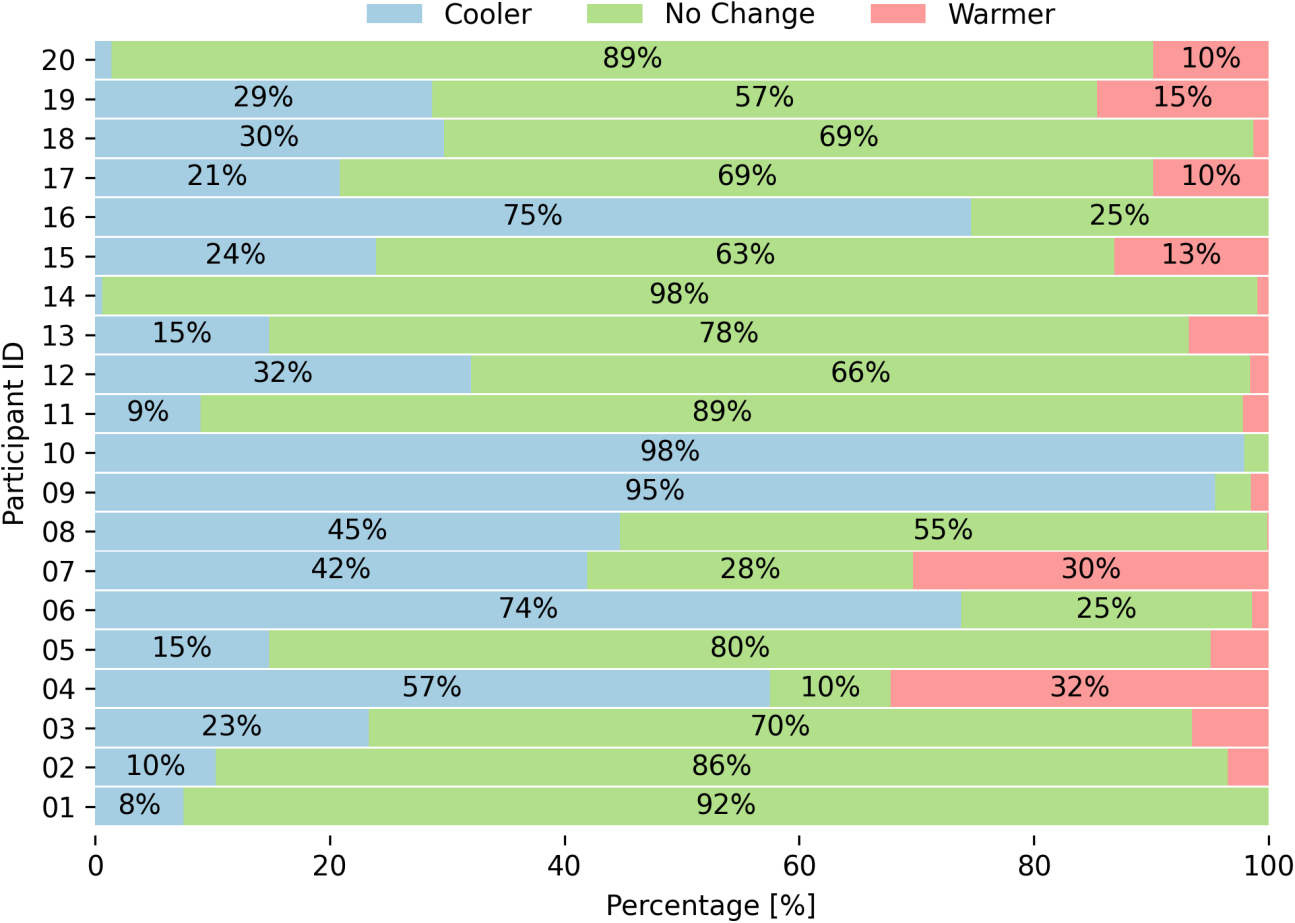
Distribution of the thermal preference responses (Q.1) provided by each participant throughout the study period.

**FIGURE 6:**
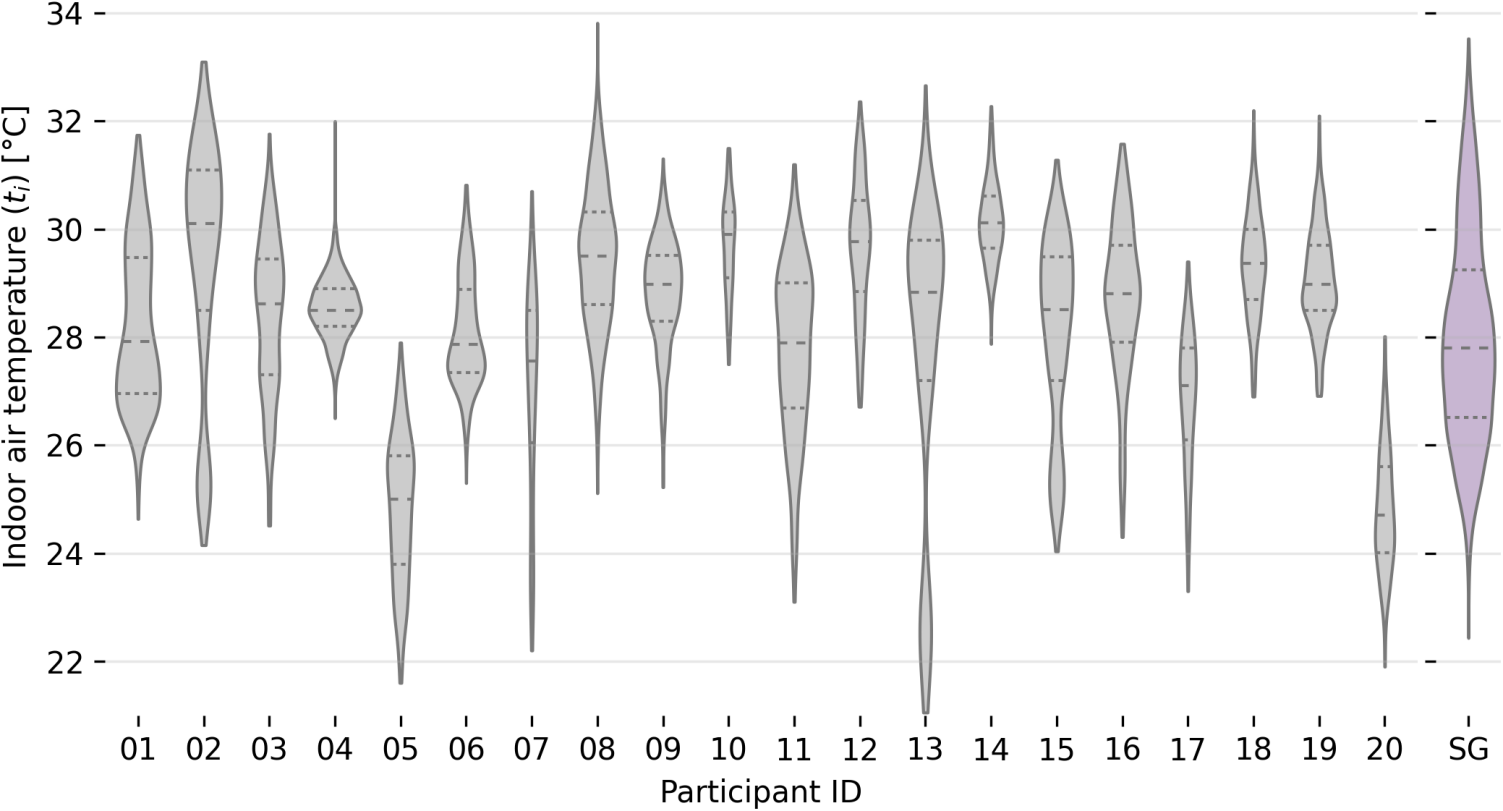
Indoor air temperature ($t_{i}$) measured when participants completed the RHRN survey. Data have been grouped by the participant. The last violin plot (purple) shows the average outdoor air temperature measured in Singapore (SG) throughout the whole duration of the study.

To better depict how participants perceive their thermal environment, in Figure 5 we plotted the distribution of the thermal preference votes (Q.1) grouped by the participant. While the great majority voted “No Change,” two wanted to be “Cooler” more than 90% of the time. Even if participants had similar distributions of thermal preference votes, such as participants 05 and 13, they might have different thermal comfort needs, requirements, and preferences. This situation can be explained by the fact that the participants wore different clothes, engaged in different activities, and were exposed to different environmental conditions. The values of $t_{i}$ recorded when a participant completed the survey are shown in Figure 6. The Figure also depicts the outdoor temperature measured in Singapore during the entire study period. Singapore is characterized by a tropically hot and humid climate with limited seasonal temperature variation. Temperature variation mainly occurs intra‐day.

**FIGURE 7:**
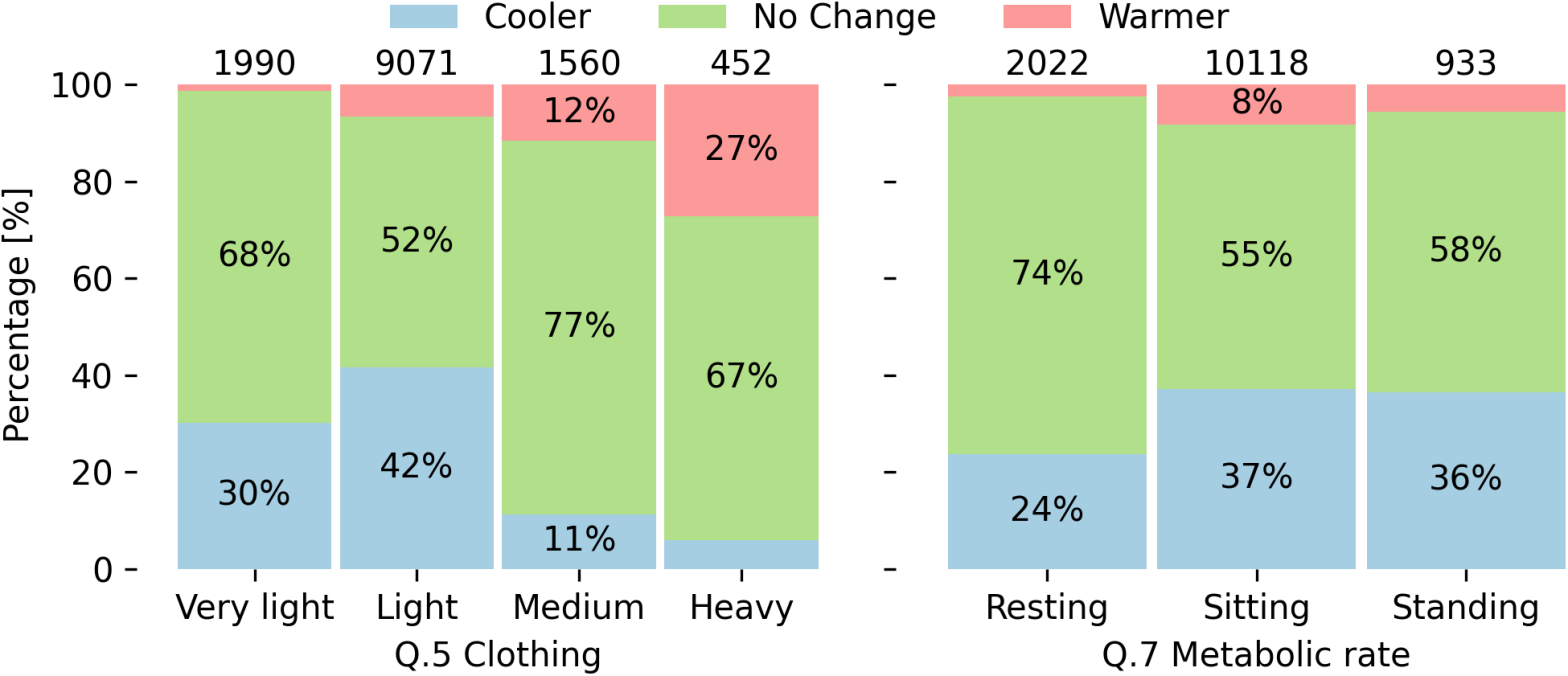
Distribution of the thermal preference responses (Q.1) provided by all participants throughout the study period grouped by their reported clothing insulation (Q.4) and metabolic rate (Q.6). The number above each bar shows the total number of responses collected for that specific answer.

The thermal preference votes grouped by the self‐reported clothing and metabolic rates are shown in Figure 7. Participants actively adjusted clothing to improve their thermal comfort. They wore “Very light” clothes to compensate for warm indoor air temperatures. Participants also actively increased their clothing levels when exposed to temperatures they deemed to be “Cold.” Thus, 67% of participants wearing “Heavy” clothing felt comfortable. Wearing more clothes alone did not always suffice to compensate for cold indoor conditions. Overcooling indoors was the leading cause that 27% of them wanted to be “Warmer,” even though participants wore “Heavy” clothing in a tropical climate. This is a common issue for buildings located in the tropics.^46^ Overcooling does not only negatively impact building energy consumption, but in the tropics has also been shown to worsen occupants' cognitive performance.^47^ Approximately 74% of the participants who reported to be “Resting” voted “No Change” in question Q.1.

### 3.3 Thermal preference personal comfort models

**FIGURE 8:**
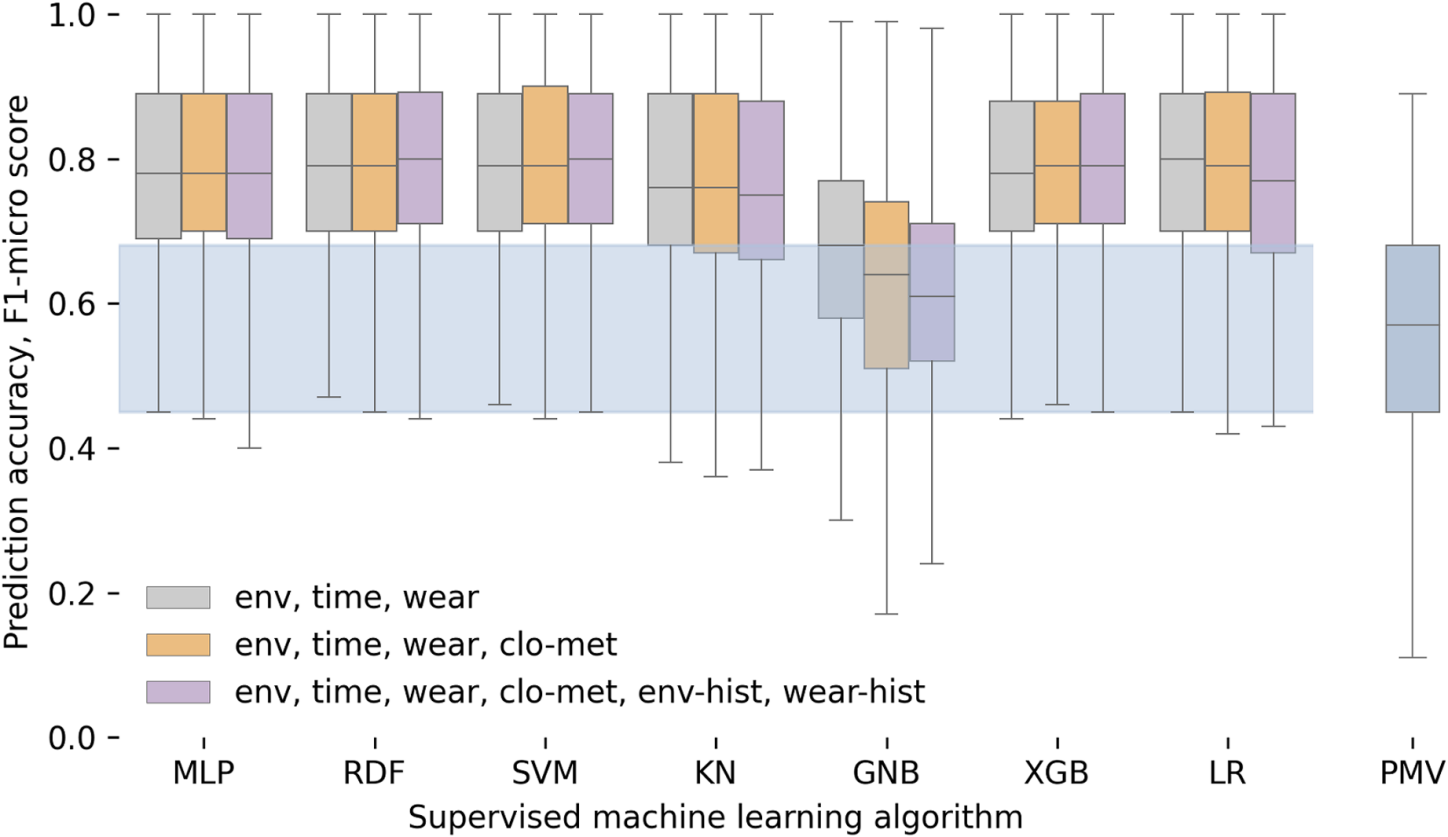
F1‐micro scores for the thermal preference personal comfort models determined using the full dataset for each participant over 100 iterations. The light blue shaded area depicts the interquartile range for the PMV model. We used the following abbreviations: MLP, Multi‐Layer Perceptron; RDF, Random Forest; SVM, Support Vector Machine; KN, K‐Nearest Neighbors; GNB, Gaussian Naive Bayes; XGB, Extreme Gradient Boosting; LR, Logistic Regression

The prediction accuracy of the personal comfort model we developed is depicted in Figure 8. The figure shows the F1‐micro scores for the three sets of variables grouped by the supervised machine learning model we used to train the personal comfort models. We also report the PMV model results.

The prediction accuracy of all the personal comfort models developed with the supervised machine learning algorithms was significantly (*p* < 0.01) and substantially (excluding XGB) higher (≈37%) than the results obtained from the PMV model. In our study, we only qualitatively logged clothing levels and metabolic rates, and we did not measure airspeed as detailed in Section 2. Hence, we do not have sufficient evidence to prove that the PMV has low prediction accuracy. We simply report the results of the PMV to provide a benchmark to show the increase in accuracy that personal comfort models can achieve. This is, however, a common issue in real buildings, hence these values must also be assumed to calculate the PMV.

One of the main objectives of this study was to determine how different sub‐sets of variables would affect the accuracy of the models. Adding an increased number of variables to the model did not always improve its accuracy. In some cases, it had the opposite effect and led to a decreased F1‐micro score. Similar results were also obtained in previous studies.^18^ This can be partially explained because participants completed surveys in near‐steady‐state conditions. Hence, including historical data is not always beneficial. Moreover, self‐reported clothing and activity may not have accurately enough represented participants' actual clothing ensembles or metabolic rates since their selection was limited to four choices. This is a positive result since in a real‐life scenario we would not have access to this information.

**FIGURE 9:**
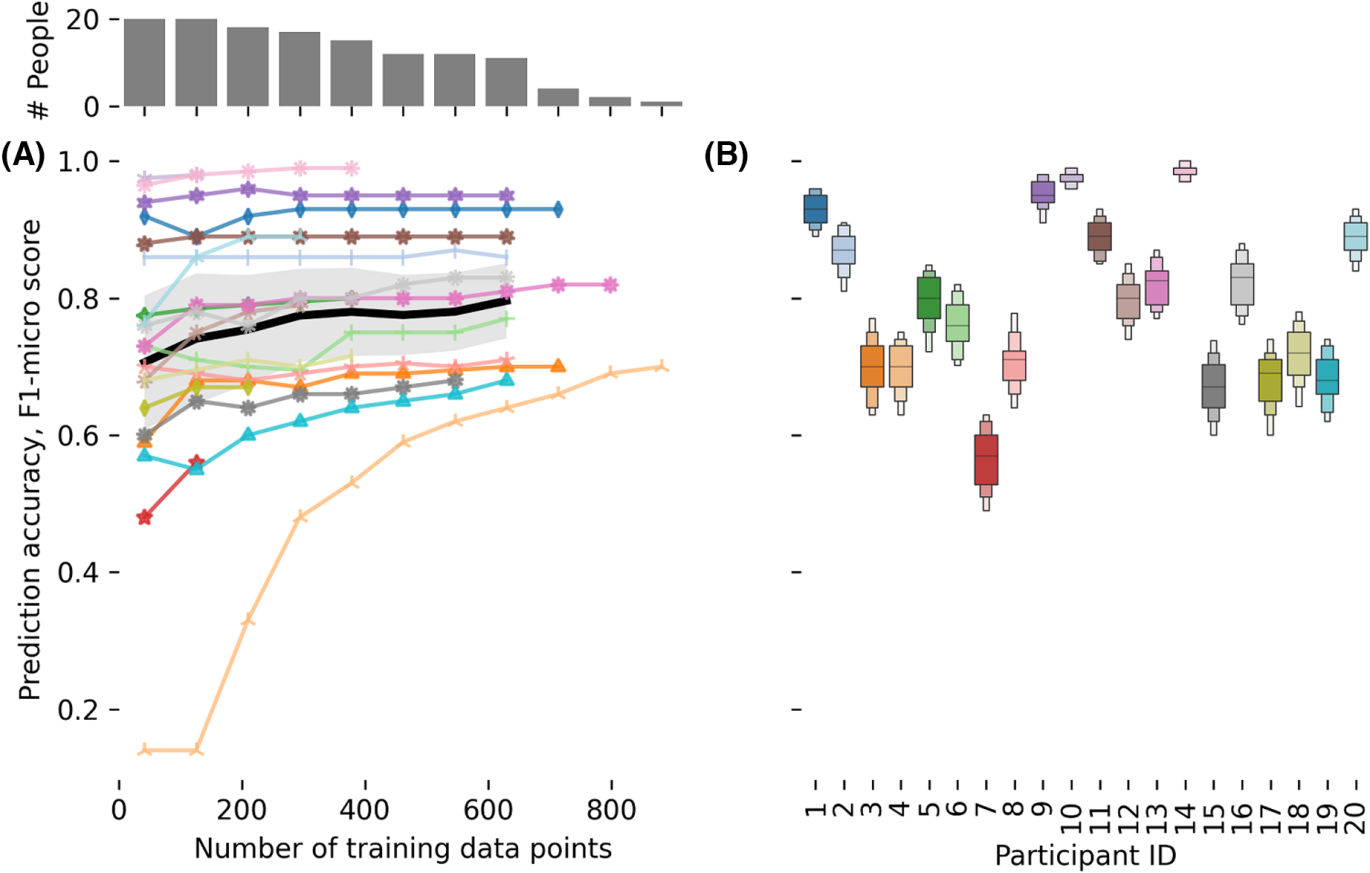
F1‐micro scores for the thermal preference personal comfort models determined using the Support Vector Machine (SVM) algorithm. (A) Shows the mean F1‐micro score for each participant, as well as the mean score (black line) and standard deviation across (shaded area) the whole study sample. The markers show the participant's mean F1‐micro scores calculated by averaging the mean scores obtained across the 100 iterations, for that specific number of training data points. A different number of valid surveys were completed by different participants. The bar plot, in (A) over the chart, shows the number of answers that were used to calculate the sample mean score and the respective standard deviation. (B) Shows all the F1‐micro scores determined using the full dataset for each participant over 100 iterations

**FIGURE 10:**
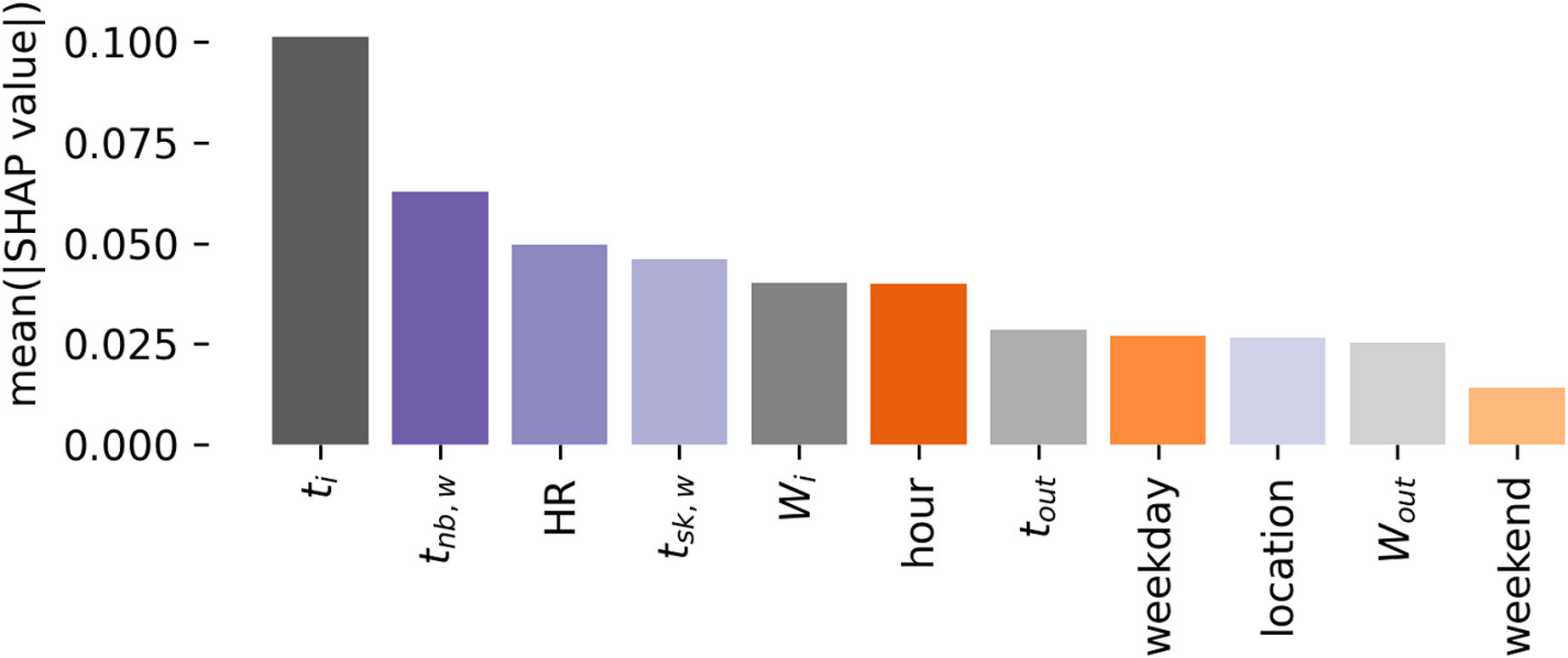
Absolute mean SHAP value of the six best‐performing supervised machine learning models. Variables are color‐coded, *environmental*—using shades of gray, *wearable*—using shades of purple, and *time*—using shades of orange. Where $t_{\text{out}}$ stands for outdoor air temperature and $W_{\text{out}}$ stands for humidity ratio outdoors.

The distribution of the F1‐micro scores was significantly different when we compared the results of the following models: XGB, SVM, RDF, LR, MLP using different variable sets. However, the significant increase in model complexity would not justify the modest increase in prediction accuracy in most practical applications. On average, training one model once with the full variable set for each 20 users resulted took 83, 6, 620, 11, and 67 s for XGB, SVM, RDF, LR, MLP models, respectively. We consequently decided to present only the results from the SVM model trained with the *environmental*—*wearable*—*time* independent sets of variables in Figures 9 and 10. Firstly, because the SVM model is less computationally intensive to train and secondly because it is a linear model, hence it is better suited to predict thermal preference which is an ordinal variable. We are providing supporting evidence on this in Section 4. Linear models use a multidimensional hyperplane to classify the data, this may lead to lower prediction accuracy if compared with non‐linear models. Nevertheless, linear models ensure that as $t_{i}$ increases, all other variables being fixed, the prediction does not switch back and forth between “Warmer,” “No Change,” and “Cooler.” This issue is particularly relevant when personal comfort models are used in real‐life applications to operate buildings. Non‐linear model predictions may be the cause of instabilities in the HVAC controller and limit the use of personal comfort models to control buildings.

#### 3.3.1 Influence of data size on prediction power

Figure 9A depicts how the F1‐micro score varies as a function of the number of training data points for each participant. The figure also shows the F1 mean score (black line) and its standard deviation (shaded area) across all participants.

The sample average accuracy mean score plateaued at around ≈300 data points. This suggests that this may be the optimal number of points we may need to collect when training personalized comfort models. It should be noted that there was high variability when the curve plateaued for each individual. This is due to the inherited differences across the personal preferences of subjects and the conditions they were exposed to. Figure 9B shows the overall accuracy of each personal comfort model over the 100 iterations. It can be observed that each personal comfort model converged to a stable value across all 100 iterations. The standard deviation of all 20 personal comfort models over all 100 iterations was similar across different participants, with a mean value of 0.035 and a standard deviation of 0.011. The same cannot be said about the overall accuracy of each personal comfort model, where the median F1 score for participant 14 was 0.99 while for participant 7 was 0.56. This, in other words, means that not all personal comfort models performed equally. Some almost always correctly predicted the thermal preference vote reported by the participants, while others had a significantly lower accuracy.

#### 3.3.2 Importance of independent variables

The absolute mean SHAP values across all six best‐performing supervised machine learning models are shown in Figure 10. Sub‐variables groups defined in Section 2.6.4 are color‐coded. While indoor air temperature ($t_{i}$), wrist near body temperature ($t_{\mathrm{nb},w}$), heart rate (*HR*), wrist skin temperature ($t_{\mathrm{sk},w}$), and humidity ratio indoors (*W*_*i*_) contributed the most to the models' final predictions, we observed a significant difference of SHAP values between different participants and across different models. In Figure A.3, we report the mean SHAP values across all participants for each supervised machine‐learning model. A detailed discussion of these results is presented in Section 4.2.

## 4 DISCUSSION

The results of our study enabled us to draw several connections to the existing literature, discuss the usefulness and limitations of the methodology and results, and motivate future work.

### 4.1 Impact of training data size on model prediction

One novel aspect of our study was the duration of the data collection, which enabled us to gather the longest longitudinal data set so far among studies that aimed to develop personal thermal comfort models.^28^ We collected more than double the amount of points per participant and we made the dataset publicly available. Personal comfort models necessitate data for both testing and training. Hence, a sufficiently large number of data points from each participant is required for the machine‐learning algorithm to converge. Figure 9 illustrates how increasing the size of trained data improves the model prediction power based on the collected data set. Across all participants, the model prediction accuracy (F1‐micro) stabilized to a plateau at around 300 data points. Individual personal models show varying degrees of sensitivity to dataset size. This insight highlights the diminishing return of collecting more than 250–300 data points for most test participants. This result is specific to our study and other authors may find a different range based on their study methodology. Our results agree and provide additional supporting evidence to validate those obtained by Liu et al.^18^ Arguably, the amount of data needed to characterize thermal comfort could be reduced even further with the development of targeted sampling that strategically requests feedback only when required to increase the model prediction power.^48^ In our study, we already implemented this strategy. Participants received a text message when exposed to environmental conditions that they rarely experienced before, to maximize the chances of obtaining a balanced dataset. However, we still asked them to complete, on average, a total of six surveys per day. This requirement can be significantly reduced or removed altogether in future studies thanks to targeted surveys. For some participants, the prediction accuracy slightly decreased as the trained data size increased from 42 to 126. This situation is expected since, as time passes, they may be exposed to a broader range of environmental factors and conditions that they did not experience before, and the model needs to learn how to predict participants' thermal preferences under these new sets of conditions. This result is a significant advantage that personal comfort models have over aggregate models since they can be re‐trained as new data are collected. This situation may be partially alleviated by the use of transfer learning, ensemble strategies, and domain adaptation which can be used to predict individual thermal preference even when there is a lack of data regarding a specific person.^49^, ^50^

We also observed that, for some participants, the F1‐micro curves did not vary much as a function of the data size (e.g., participants 9 and 10). Some possible causes of this are that participants were constantly exposed to warm temperatures and that some did not maintain compliance with experimental guidelines. The latter point is discussed in Section 4.3. For example, participant 10 was always exposed to temperatures above 27.5°C when completing the RHRN survey and reported wanting to be “cooler” 98% of the time. This scenario is expected in Singapore, where the recorded outdoor temperature over the 6‐month study period was higher than 26.5°C for 75% of the time.

### 4.2 Independent variables' importance in thermal preference prediction

We used SHAP values to quantify of the impact that each independent variable had on the accuracy of the personal models. While the average magnitude for each variable varied in different models indoor air temperature ($t_{i}$), wrist near body temperature ($t_{\mathrm{nb},w}$), heart rate (*HR*), wrist skin temperature ($t_{\mathrm{sk},w}$), and humidity ratio indoors (*W*_*i*_) contributed the most to the models' final predictions. This insight is in line with the existing body of knowledge since $t_{i}$ is the primary driver of sensible heat loss or gain from the environment to the human body. Our results reinforce previous work.^18^ The *HR* is a proxy for the level of activity of the person, and it is positively correlated with the metabolic rate. The value of $t_{\mathrm{sk},w}$ reflects the vasomotor tone. The human body uses vasoconstriction and vasodilation for thermoregulation.^15^ Finally, *W*_*i*_ influences the latent heat loss toward the environment. On the other hand, the outdoor air temperature, occupant location, and outdoor humidity ratio only had a marginal contribution to the final prediction, which can be explained by the fact that these variables do not directly influence people's thermal sensation or preference, in particular during steady‐state conditions. The value of the outdoor air temperature only indirectly affects occupants' thermal preferences since they may influence the type of clothing that participants decide to wear before leaving their homes. This result may, however, only be applicable to climates similar to the one in Singapore that are characterized by limited variability.

#### 4.2.1 Self‐reported clothing and activity

We found that including self‐reported clothing and activity in some models did not significantly increment the model prediction accuracy. While this seems to be counterintuitive since both clothing and metabolic rate play a significant role in human thermoregulation, we believed that they did not increase the model prediction accuracy since they were reported qualitatively by participants who only had four options to choose from. Other measured variables like *HR* may better correlate with the participant's actual metabolic rate than self‐reported activity. This result has positive implications since, in a real‐world application, the building controller would not have access to information about clothing and activity levels.

#### 4.2.2 Near‐body temperature

While our results showed that $t_{\mathrm{nb},w}$ significantly contributed to the model prediction, it should be noted that $t_{\mathrm{nb},w}$ was strongly correlated with both $t_{i}$ and $t_{\mathrm{sk},w}$. Consequently, it would be sufficient to measure these two latter variables in most cases. On the other hand, only using $t_{\mathrm{nb},w}$ as a proxy for $t_{i}$ would decrease the complexity of the data collection, but at the same time, it would reduce the overall model accuracy. We decided to measure, log, and include in the models $t_{\mathrm{nb},w}$ since many people in warm climates use fans to cool themselves. Measuring airspeed in the proximity of the occupants in longitudinal studies is impractical, very expensive, and inaccurate. Battery‐powered anemometers would need to be recharged frequently, are very expensive, and are sensitive to direction. Airspeed varies significantly both spatially and temporally; consequently, accurate readings can only be obtained in laboratories using scientific‐grade sensors installed on stands mounted near the subject. The value of wrist near body temperature can then be used as a proxy to partially compensate for the lack of airspeed data. When airspeed is low, $t_{\mathrm{nb},w}$ is significantly affected by the thermal plume of the participant and in turn by $t_{\mathrm{sk}}$_._ ^51^ On the other hand, when participants are cooling themselves using electric fans, the airflow disrupts the thermal plume, and $t_{\mathrm{nb},w}$ is mainly influenced by $t_{i}$.

#### 4.2.3 Skin temperature

Participants did not report any significant discomfort by wearing the iButton for an extended period. At the end of the study, 16 participants answered positively to the following question: “Would you wear the Fitbit and complete a few surveys per day for two weeks for no financial reward, if you knew that the information would improve your well‐being indoors?” However, measuring $t_{\mathrm{sk},w}$ using an iButton adds complexity and maybe still a source of mild discomfort for some people. iButton cannot communicate wirelessly; hence data cannot be accessed in real time. There have been several announcements from the leading smartwatch manufacturers to include a skin temperature sensor in their devices. Still, at the time of writing this manuscript, no smartwatch available on the market could measure it accurately. However, in September 2022 at the time of reviewing this manuscript, Apple announced that they have released a new Apple Watch that can accurately measure skin temperature.

#### 4.2.4 Historical variables

The increases in model accuracy when historical variables were added to the model did not justify the increased complexity. This situation can be partially explained by the fact that we carefully chose to analyze data collected when participants were in near “steady‐state” conditions. This choice was driven by the fact that people in their office, on average, spend most of their time at their desks in near “steady‐state” conditions. Predicting how people perceive their thermal environment during transitory conditions goes beyond the scope of our research.

### 4.3 The compliance rate of participants and data quality considerations

Six months of the daily longitudinal collection is a challenge in terms of ensuring that participants maintain compliance with experimental guidelines. The Cozie smartwatch‐based methodology turned out to facilitate high compliance with none of the participants dropping out from the study, and all completed at least 1080 surveys. This result reinforces previous work in micro‐EMA and its ease of deployment in collecting longitudinal data with less survey fatigue.^32^ Compliance maintenance was enhanced with notifications sent through a messaging app that would remind the participants about notable achievements or deficiencies in the experimental process.

Despite the compliance rate, some participants were not fully cognizant of their perspective on each response given over the 6 months due to survey fatigue. This risk could be mitigated in future work through early detection, incentives, and by significantly reducing the number of surveys that each participant has to complete every week. This risk is significant for data‐driven models, which are highly susceptible to “bad” data. One possible other solution to this problem is utilizing the model to control their environment actively.

### 4.4 Limitations

One notable limitation of the deployment is that the Singapore climate has little diversity across the year. Seasonality in other climates may result in longitudinal data needing more training beyond the 200–300 points found in this study. Studies in other climates may need to spread data collection into phases that account for different seasons.

In addition, the experimental deployment for this study began in April 2020, just as Singapore entered a lockdown period due to COVID‐19 restrictions. Throughout the study, the lockdown situation was dynamic, but overall there was less diversity of data collection locations than intended. Most of the occupants were forced to work from home for the whole duration of the study, while those who were allowed to resume going to the office were required to wear face masks at all times. We started this study before the pandemic started, hence we did not include any questions about face masks.

Another notable limitation category relates to the nature of black‐box machine learning models in the application of thermal comfort prediction. The lack of conversion of model output or accuracy into the physical understanding of what makes people feel comfortable or not is troublesome in the context of improving comfort, particularly for facility operators. Future work should focus on the conversion of the accuracy of prediction to the applicability to system and occupant interaction. The previously mentioned personal comfort review found similar insight in the literature of such models.^28^ Among the different models tested, Random Forest is one of the most widely adopted in the literature and its performance justifies its adoption (Figure 8). Nevertheless, when compared to a regression‐based model like SVM with similar prediction performance, Random Forest required 100 times more computational time for model training, i.e., 620 and 6 s, respectively. Coincidentally, XGB and MLP also achieve a similar performance but require roughly 12 times the computational time of SVM, 83 and 67 s, respectively. These results reinforce the selection of SVM since it does not sacrifice prediction accuracy; as a regression‐based model, it is more interpretable and requires less computational cost. It should also be noted that since some machine learning models are not linear, like RDF, this may cause the personal comfort model may still predict thermal preference to vary back and forward from “warmer” to “cooler” as the temperature increases, despite all other inputs being fixed. This situation has several issues. Firstly, it does not provide an accurate representation of how people perceive their thermal environment nor take into account that thermal preference is an ordinal variable. Secondly, it may be the cause of instabilities if the model is used to actively control a space. We believe that this issue has had very little coverage in previous studies that aimed to develop personal thermal comfort models, and it should be further investigated.

## 5 CONCLUSIONS

We conducted a longitudinal thermal comfort study that aimed to develop personal thermal comfort models. Twenty participants took part in it, and they completed on average at least six RHRN surveys per day for a period of 6 months. We developed an effective methodology that simplified the life of the participants, and none of them dropped from the study. We measured and logged environmental parameters, physiological signals, outdoor weather data, and participants' location outdoors and indoors. We used these data to train and test a personal thermal comfort model for each participant. We were able to determine that:

- Cozie, a micro‐EMA open‐source Fitbit and Apple application, is a reliable and robust solution to non‐intrusively collect participants’ feedback in field studies.
- Personal comfort models were able to accurately predict (median F1‐micro score 0.78) occupants’ thermal preferences. With the limitations in data collection posed by the study methodology, they could outperform the PMV model.
- Indoor air temperature ($t_{i}$), wrist near body temperature ($t_{\mathrm{nb},w}$), heart rate (*HR*), wrist skin temperature ($t_{\mathrm{sk},w}$), and humidity ratio indoors (*W*_*i*_), listed in decreasing order of importance, had the highest average marginal contribution to the overall model prediction.
- The thermal personal comfort model prediction accuracy (F1‐micro) plateaued at around 300 data points across all participants. Individual personal models are sensitive to dataset size to varying degrees. The amount of data required to characterize thermal comfort could potentially be reduced with the development of targeted sampling, which strategically requests feedback only when it is necessary.
- We made available publicly the data we collected and open‐sourced the Python code we used to analyze them to enable other researchers to test different hypotheses utilizing our data.

## Nomenclature

*HR*
: heart rate, beats per minute
PMV: Predicted Mean Vote
RHRN: Right‐Here‐Right‐Now
SVM: Support Vector Machine
*t*_*i*_
: indoor air temperature, °C
*t*_*nb*,*w*_
: wrist near body temperature, °C
*t*_*sk*_
: skin temperature, °C
*t*_*sk,w*_
: wrist skin temperature, °C
*W*_*i*_
: humidity ratio indoors, kg^water vapor^/kg^dry air^

## FUNDING INFORMATION

This research has been supported by the Republic of Singapore's National Research Foundation through a grant to the Berkeley Education Alliance for Research in Singapore (BEARS) for the Singapore‐Berkeley Building Efficiency and Sustainability in the Tropics (SinBerBEST) Program.

## CONFLICT OF INTEREST

The authors declare that they have no known competing financial interests or personal relationships that could have appeared to influence the work reported in this paper.

## Supporting information


Appendix S1

## Data Availability

The source code we used to analyze the data and the full dataset are publicly available at this URL: https://github.com/FedericoTartarini/dorn‐longitudinal‐tc‐study.
