# Supplementary material for: Personal comfort models based on a 6‐month experiment using environmental parameters and data from wearables
Source: Indoor Air. 2022 Nov 25;32(11):e13160. doi: 10.1111/ina.13160 (PMC9827859; doi:10.1111/ina.13160)
Supplement: Supplementary file 1 — Appendix S1 [file INA-32-0-s001.pdf]

1 Appendix

Nomenclature

HR	heart rate, beats per minute
RHRN	Right-Here-Right-Now
SVM	Support Vector Machine
t_i	indoor air temperature, °C
$t_{nb,w}$	wrist near body temperature, °C
t_{sk}	skin temperature, °C
$t_{sk,w}$	wrist skin temperature, °C

A Methodology

A.1 Sensors

We used Fitbit Versa smartwatches since they are capable of accurately continuously tracking heart rate (HR) [1] and have a touch screen that can be used to complete Right-Here-Right-Now (RHRN) surveys using the Cozie clock-face.

iButtons are wireless temperature sensors that can accurately measure and log skin temperature (t_{sk}) [2]. We selected a 180 s sampling period, hence each iButton could record data for approximately 127 days. A sampling period of 180 s provides reasonably accurate results while limiting to two the number of times that data had to be downloaded throughout our study [3].

Participants were asked to install Netatmo Smart Home Weather Station weather stations (NWS03, Netatmo, France) in the room of their house, where they spent the majority of their time indoors. The indoor module was used to monitor indoor air temperature (t_i) and relative humidity.

A UbiBot WS1 Pro (UbiBot, China) was used to measure and log t_i and relative humidity at the participant's workplace. Participants installed the UbiBot on their work desk far away from heat sources.

Each participant also installed an iButton (model DS1925) on a bag/backpack of their choice.

Table A.1: Sensors specifications.

Manufacturer - Product name	Location	Variables Measured	Accuracy Manufacturer	Range
Fitbit – Versa	Non-dominant wrist	HR	Not reported 91 % [1]	Not reported
Netatmo – Smart Home Weather Station	Home	t_i<br>RH	±0.3°C<br>±3 %	0 to 50 °C<br>0 to 100 %
UbiBot – WS1 Pro	Work desk	t_i<br>RH	±0.3°C<br>±3 %	-20 to 60 °C<br>1 to 90 %
iButton – DS1925	Inner wrist	t_{sk}	±0.5°C	-20 to 85 °C
iButton – DS1925	Smarwatch wristband	wrist near body temperature ($t_{nb,w}$)	±0.5°C	-20 to 85 °C
iButton – DS1925	Bag	t_i	±0.5°C	-20 to 85 °C

A.2 Data Analysis

The rationale behind our decision to exclude some of the data collected as stated in Section 2.6.1 is exaplained below. During exercise, metabolic rate and the body's ability to dissipate heat towards the environment varies extensively among people based on various factors (i.e., weight, age, intensity, and fitness level). Hence, we excluded surveys completed while the participant was exercising. Evaluating thermal comfort preference during transitories requires detailed knowledge of all the environmental conditions that occupants were exposed to during and before the event. Moreover iButtons may not be suited to measure t_{sk} in these conditions accurately. Finally, we excluded all the surveys completed when the $t_{nb,w}$ was at least 1 °C higher than the $t_{sk,w}$. Throughout the data collection period, the

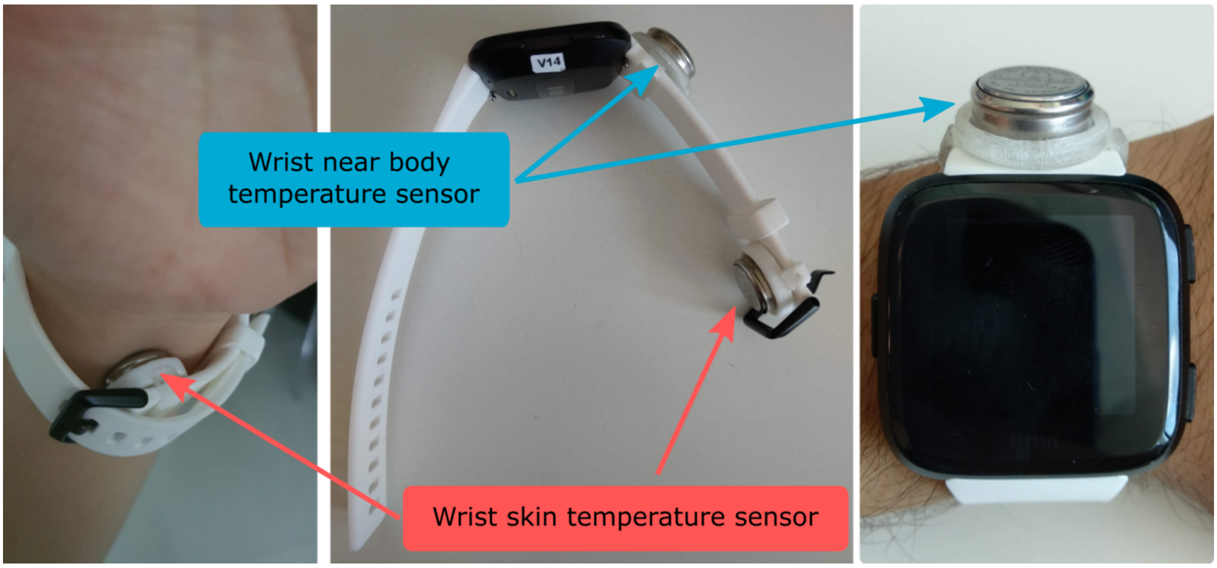

Figure A.1: Fitbit Versa v1 and the two iButtons we used in this study to measure wrist skin temperature ($t_{sk,w}$) and $t_{nb,w}$.

outdoor temperature never exceeded 34 °C consequently, a value of $t_{nb,w}$ equal or higher than $t_{sk,w}$ can be explained mainly by one of the following conditions: the participant did not wear the smartwatch as recommended, and consequently, the sensor was not in good contact with the skin and its readings were significantly influenced by t_i surrounding the occupant; the sensor was in contact with another body part, and measured the skin temperature of another body location; direct solar radiation heated the sensor. We also filter out all the surveys completed in less than 3.2 s. This decision was taken before conducting the field study since we foresaw that some participants may have intentionally tried to take advantage of the study to get the final compensation. This threshold was the calculated average response speed we obtained by completing 50 surveys as fast as possible. Entries in the dataset with missing values were dropped from the data analysis.

We analyzed the data using Python v3.8 [4]. We also used the following Python Packages to perform the analysis: pandas [5], scikit-learn [6], Matplotlib [7], seaborn [8], pythermalcomfort [9], xgboost [10], PsychroLib [11], numpy [12], and shap [13]. The lines in the violin plots show the 1st, 2nd (median), and 3rd quartiles.

References

[1] Benjamin W Nelson and Nicholas B Allen. "Accuracy of Consumer Wearable Heart Rate Measurement During an Ecologically Valid 24-Hour Period: Intraindividual Validation Study". In: *JMIR mHealth and uHealth* 7.3 (Mar. 2019), e10828. ISSN: 2291-5222. DOI: `10.2196/10828`. URL: `https://mhealth.jmir.org/2019/3/e10828/`.

[2] Wouter D. van Marken Lichtenbelt et al. "Evaluation of wireless determination of skin temperature using iButtons". In: *Physiology and Behavior* 88.4-5 (2006), pp. 489–497. ISSN: 00319384. DOI: `10.1016/j.physbeh.2006.04.026`.

[3] Federico Tartarini and Stefano Schiavon. "Skin temperature sampling period for longitudinal thermal comfort studies". In: *16th Conference of the International Society of Indoor Air Quality and Climate: Creative and Smart Solutions for Better Built Environments, Indoor Air 2020*. 2020, pp. 1–20. ISBN: 9781713823605. DOI: `https://doi.org/10.5281/zenodo.4289851`.

[4] G. van Rossum. *Python tutorial*. Tech. rep. CS-R9526. Amsterdam: Centrum voor Wiskunde en Informatica (CWI), 1995.

[5] The pandas development team. *pandas-dev/pandas: Pandas*. Version latest. Feb. 2020. DOI: `10.5281/zenodo.3509134`.

[6] F. Pedregosa et al. "Scikit-learn: Machine Learning in Python". In: *Journal of Machine Learning Research* 12 (2011), pp. 2825–2830.

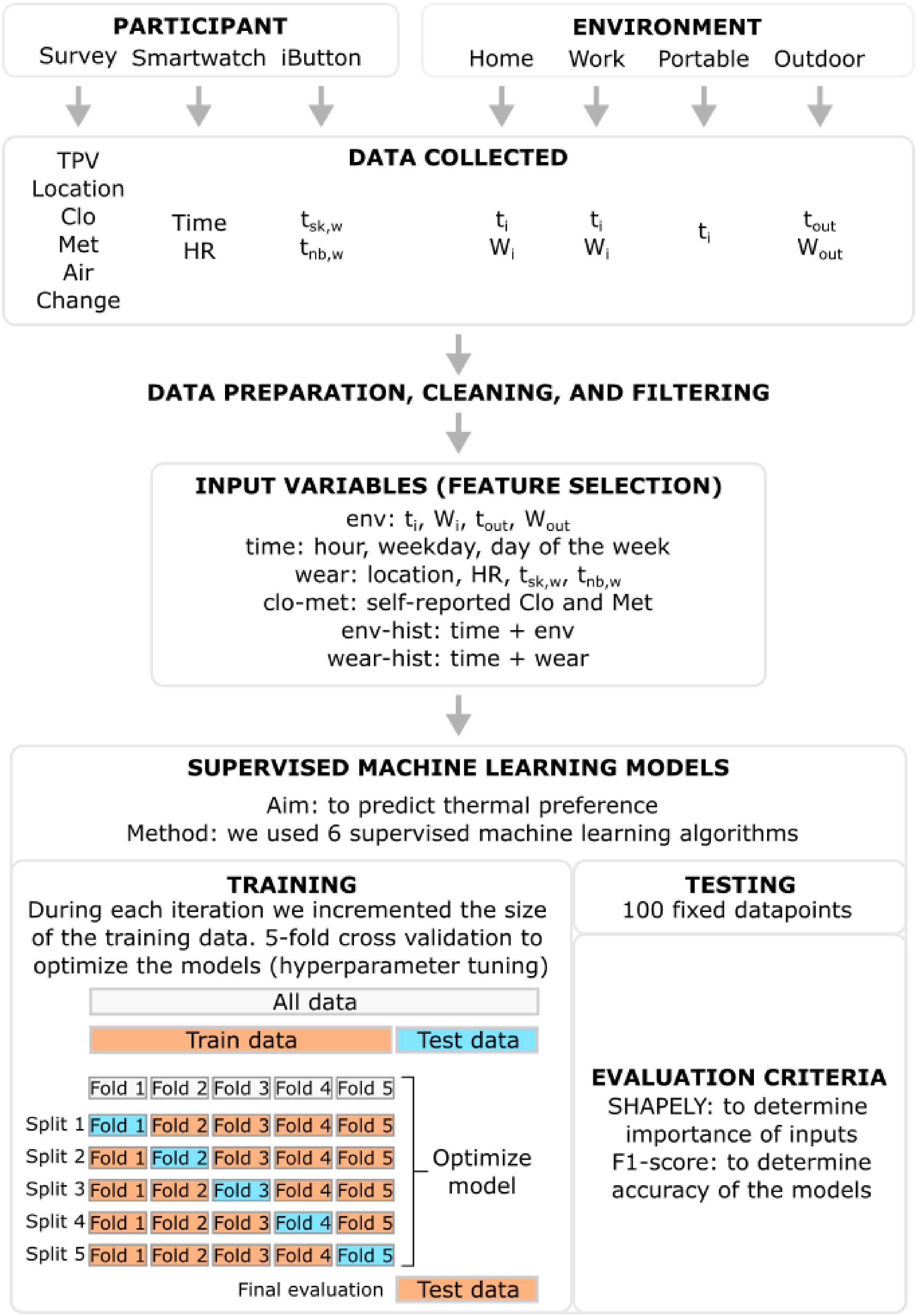

Figure A.2: Flowchart depicting the methodology used used to collect and analyze the data in our study.

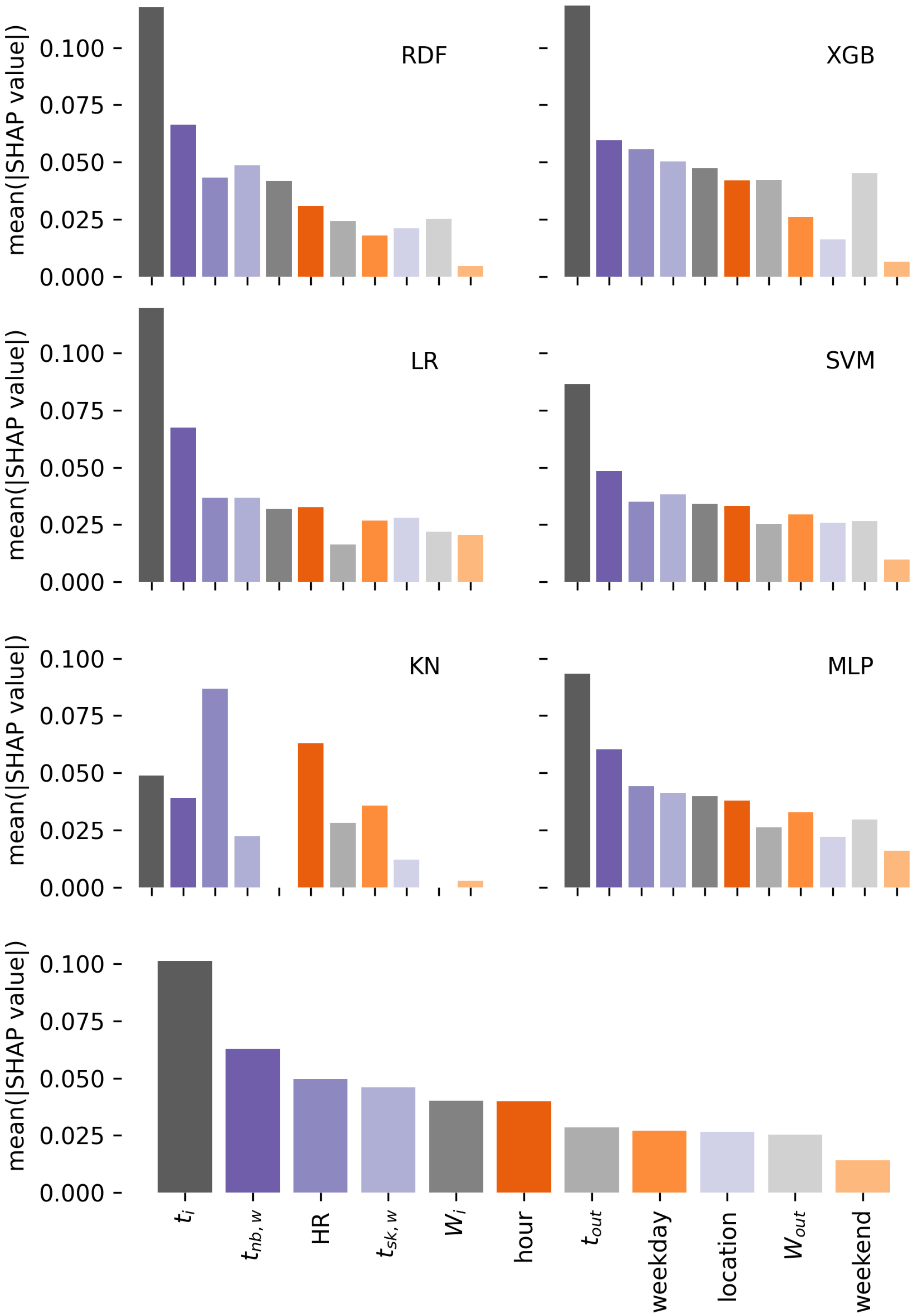

Figure A.3: The SHAP values of the six best performing supervised machine learning models are shown in the top six Figures, while the bottom Figure shows the mean SHAP values of all the six above models. Variables are color coded, *environmental* – using shades of gray, *wearable* – using shades of purple, and *time* – using shades of orange. Where t_{out} stands for outdoor air temperature, and W_{out} stands for humidity ratio outdoors.

Table A.2: Hyper-parameters used in the grid search.

Model	Value
Random Forest	Number of trees: 100, 300, 500<br>Split criterion: Gini index<br>Min. samples for split: 2, 3, 4<br>Min. samples on an edge : 1, 2, 3<br>Class weight : balanced
Logistic Regression	Inverse of regularization strength: 0.01, 0.1, 1, 10, 100<br>Fit intercept: True, False
Extreme Gradient Boosting	Number of gradient boosted trees: 100, 300, 500<br>Maximum tree depth: 2, 4, 6, 8, 10
Support Vector Machine (SVM)	Regularization parameter: 0.01, 0.1, 1, 10, 100<br>Kernel: rbf, sigmoid
K-Nearest Neighbors	Number of neighbors: 1, 2, 3, 5, 7<br>Weights: uniform, distance
Gaussian Naive Bayes	-
Multi-Layer Perceptron	Hidden layer size: (13, 13)

[7] J. D. Hunter. "Matplotlib: A 2D graphics environment". In: *Computing in Science & Engineering* 9.3 (2007), pp. 90–95. DOI: 10.1109/MCSE.2007.55.

[8] Michael Waskom and the seaborn development team. *mwaskom/seaborn*. Version latest. Sept. 2020. DOI: 10.5281/zenodo.592845.

[9] Federico Tartarini and Stefano Schiavon. "pythermalcomfort: A Python package for thermal comfort research". In: *SoftwareX* 12 (July 2020), p. 100578. ISSN: 23527110. DOI: 10.1016/j.softx.2020.100578.

[10] Tianqi Chen and Carlos Guestrin. "XGBoost: A Scalable Tree Boosting System". In: *Proceedings of the 22nd ACM SIGKDD International Conference on Knowledge Discovery and Data Mining*. KDD '16. San Francisco, California, USA: ACM, 2016, pp. 785–794. ISBN: 978-1-4503-4232-2. DOI: 10.1145/2939672.2939785.

[11] D. Meyer and D. Thevenard. "PsychroLib: a library of psychrometric functions to calculate thermodynamic properties of air". In: *Journal of Open Source Software* 4.33 (2019), p. 1137. DOI: 10.21105/joss.01137.

[12] Charles R. Harris et al. "Array programming with NumPy". In: *Nature* 585.7825 (Sept. 2020), pp. 357–362. DOI: 10.1038/s41586-020-2649-2.

[13] Scott M Lundberg and Su-In Lee. "A Unified Approach to Interpreting Model Predictions". In: *Advances in Neural Information Processing Systems 30*. Ed. by I. Guyon et al. Curran Associates, Inc., 2017, pp. 4765–4774.
